# Trimethoprim/sulfamethoxazole and risk of haemophagocytic lymphohistiocytosis (HLH): a literature review and disproportionality analysis using individual case safety reports from FAERS

**DOI:** 10.1093/jac/dkaf231

**Published:** 2025-07-15

**Authors:** Rinko Lau, Hasti Sadeghi, Fatemeh Ahmadi, Niaz Chalabianloo, Rebecca Preyra, Mohammad Ali Omrani, Flory T Muanda

**Affiliations:** Department of Physiology and Pharmacology, Western University, London, ON, Canada; Department of Biology, Western University, London, ON, Canada; Department of Epidemiology and Biostatistics, Western University, London, ON, Canada; ICES Western, Kidney Dialysis and Transplantation Research Program, London, ON, Canada; Department of Physiology and Pharmacology, Western University, London, ON, Canada; Department of Physiology and Pharmacology, Western University, London, ON, Canada; Department of Physiology and Pharmacology, Western University, London, ON, Canada; Department of Physiology and Pharmacology, Western University, London, ON, Canada; Department of Epidemiology and Biostatistics, Western University, London, ON, Canada; ICES Western, Kidney Dialysis and Transplantation Research Program, London, ON, Canada; Lawson Health Research Institute, London Health Sciences Centre, London, ON, Canada

## Abstract

**Objectives:**

This study aimed to review existing literature regarding the association between haemophagocytic lymphohistiocytosis (HLH) and trimethoprim/sulfamethoxazole and examine the risk of HLH using data from the FDA Adverse Event Reporting System (FAERS).

**Methods:**

We conducted a literature review to identify studies investigating the link between trimethoprim/sulfamethoxazole and HLH. Additionally, we performed an active-comparator restricted disproportionality analysis (ACR-DA) in FAERS (2004Q1–2023Q4) for trimethoprim/sulfamethoxazole compared to amoxicillin/clavulanate and azithromycin. We calculated reporting odds ratios (ROR), adjusted ROR (accounting for age category and indications for respiratory and urinary tract infections), proportional reporting ratios and information components (IC_25_), to identify potential safety signals for HLH.

**Results:**

Our literature review identified five case reports of HLH linked to trimethoprim/sulfamethoxazole, showing a male predominance of 80% and a median age of 39 years. Symptoms appeared 2 to 14 days after initiating treatment, with fever being the first symptom reported in all patients. The disproportionality analysis indicated that reports of HLH associated with trimethoprim/sulfamethoxazole were approximately 3 to 14 times more frequent than those for amoxicillin/clavulanate (ROR, 3.08; 95% CI, 1.67–5.68) and azithromycin (ROR, 13.89; 95% CI, 5.88–32.82), with most reports submitted by other healthcare professionals.

**Conclusions:**

Trimethoprim/sulfamethoxazole may be linked to an increased risk of HLH. Further pharmacoepidemiologic research is necessary to confirm these findings and inform the safe clinical use of trimethoprim/sulfamethoxazole.

## Introduction

Haemophagocytic lymphohistiocytosis (HLH) is a rare but potentially life-threatening condition characterised by excessive immune activation and the production of pro-inflammatory cytokines.^1^ This hyperinflammatory response can lead to haemophagocytosis, multi-organ failure, and, in severe cases, death.^2^ The hallmark clinical features of HLH include high fever, cytopenia, rash and hyperferritinemia.^3^ HLH is classified into two subtypes: primary (familial), which is linked to specific genetic mutations and primarily affects children, and secondary (acquired), which arises from a variety of underlying conditions, including infections, neoplastic diseases and the use of certain medications.^4^

Trimethoprim/sulfamethoxazole is a popular prescribed antibiotic combination that treats and prevents several bacterial infections, including urinary tract, respiratory and skin infections.^5^ Although trimethoprim/sulfamethoxazole is generally well-tolerated, it has been associated with several adverse effects (AEs), including myelosuppression, hepatotoxicity, acute renal damage, lung toxicity and gastrointestinal complications.^6,7^ Notably, case reports have suggested a probable link between trimethoprim/sulfamethoxazole and drug-induced HLH.^8^

In 2023, Health Canada conducted a safety review of HLH associated with trimethoprim/sulfamethoxazole using data from the Canada Vigilance database, international sources and scientific literature. Among 10 international cases reviewed, 1 was probably linked to the drug, 8 were possibly linked (with confounding medical conditions), and 1 was unlikely to be linked. Two deaths were reported: one possibly linked to the drug and the other unlikely to be related.^9^ These findings prompted Health Canada to issue a warning about the potential risk of HLH associated with trimethoprim/sulfamethoxazole use and call for further investigation to confirm this AE. However, no research has yet studied trimethoprim/sulfamethoxazole and its potential link to HLH utilizing data from the US Food and Drug Administration’s Adverse Event Reporting System (FAERS) or other large individual case safety report databases.

To address this gap, we comprehensively reviewed the published literature and performed an active-comparator restricted disproportionality analysis (ACR-DA) on data from the FAERS. Our objective was to assess the existing evidence on the potent trimethoprim/sulfamethoxazole and HLH, and to investigate this association further through ACR-DA. We hypothesize that trimethoprim/sulfamethoxazole is associated with an increased risk of HLH.

## Methods

### Literature review

We systematically searched Medline and Embase databases to identify relevant literature published up to 1 March 2025. The search strategy included various keywords related to trimethoprim/sulfamethoxazole and HLH (Table S1, available as Supplementary data at JAC Online). We included randomized controlled trials, cohort studies, case-control studies, cross-sectional studies, case series and case reports.

The review was conducted by two independent reviewers (R.L. and H.S.), who screened the titles and abstracts of identified articles for relevance. Articles that did not meet the predetermined inclusion criteria were excluded. Full texts of the remaining articles were reviewed to assess eligibility. A standardized data extraction form was used for all included reports to ensure consistency. Extracted data included information on authors, publication year, study design, patient demographics (age and sex), indication for trimethoprim/sulfamethoxazole use, dosage, time to onset of AEs and clinical outcomes.

Only English-language publications were considered, and we excluded review articles, *in vitro* studies, and studies lacking full-text availability. The Naranjo Adverse Drug Reaction Probability Scale was applied to all case reports to assess the likelihood of HLH being associated with trimethoprim/sulfamethoxazole use.

### DA using ICSRs from FAERS

An ACR-DA was conducted using Individual Case Safety Reports (ICSRs) from the FAERS database.^10^ This method, a specialized form of DA, is commonly used to identify safety signals. Unlike traditional DA, which compares the safety outcomes of a specific medication with all other medications in the database, ACR-DA focuses on comparing the safety outcomes of the medication of interest, in this case, trimethoprim/sulfamethoxazole, with those of an active comparator sharing similar indications.^11^ This targeted approach helps reduce the risk of false-positive signals. As such, we compared HLH reports for trimethoprim/sulfamethoxazole with those for two other antibiotics, amoxicillin/clavulanate and azithromycin, which are commonly used for similar indications, even though these antibiotics have different mechanisms of action. Our reporting follows the Reporting of a Disproportionality Analysis for Drug Safety Signal Detection Using Individual Case Safety Reports in Pharmacovigilance (READUSPV) (Tables S2 and S3).^12^

## FAERS database

### Data source

This study utilized data from the FAERS, accessed via the OpenFDA platform.^13,14^ The dataset spans from the first quarter of 2004 to the fourth quarter of 2023. OpenFDA was chosen due to its pre-processed format, which reduces redundancies and provides a more structured dataset, ensuring efficient analysis compared with raw FAERS data.^15^

### Data extraction, cleaning and processing

Data extraction was accomplished through a tailored script designed to download JSON files relevant to our study period. While the structured format provided by OpenFDA was beneficial, we encountered numerous inconsistencies in drug names, particularly within the ‘medicinal product’ field, necessitating extensive data cleaning. Our manual review uncovered a variety of drug name variations, which ultimately enriched the dataset. To address this, we utilized regular expressions to standardize these names during the preprocessing stage and aligned them with established databases such as RxNorm and Observational Health Data Sciences and Informatics (OHDSI).^16^

We integrated RxNorm Concept Unique Identifiers (RxCUIs), generic drug names and codes from the Anatomical Therapeutic Chemical (ATC) classification system to further augment our dataset.^17^ We also mapped the drug characterization fields from OpenFDA to the role code field in FAERS, aiding in identifying primary suspect drugs. Additionally, we identified and removed duplicate cases by cross-referencing safety report ID numbers tied to each report, ensuring our final dataset was clean and focused on the primary suspects of adverse reactions.

### Exposed and comparator groups

The exposed cohort consisted of ICSRs where trimethoprim/sulfamethoxazole was identified as the primary suspect for the AE. Two comparator groups were defined: one for amoxicillin/clavulanate and another for azithromycin. These comparators were selected based on their comparable clinical indications to trimethoprim/sulfamethoxazole.

### Study outcome

We used the following Medical Dictionary for Regulatory Activities (MedDRA) preferred terms—haemophagocytic lymphohistiocytosis (HLH)—to capture the study outcome.

### Statistical analysis

ACR-DA was conducted to evaluate the association between trimethoprim/sulfamethoxazole and HLH using both frequentist and Bayesian statistical methods. The frequentist analysis employed the proportional reporting ratio (PRR) and reporting odds ratio (ROR), which compared the incidence of HLH associated with trimethoprim/sulfamethoxazole to other medications in the dataset. Bayesian analysis was performed using the information component (IC), which identifies disproportionality by calculating the deviation between observed and expected event rates for specific drug-event pairs. A signal for disproportionality was defined based on three criteria: (i) an IC_25_ greater than 0, indicating significant disproportionality; (ii) the lower bound of the 95% confidence interval for the ROR and PRR exceeding 1.0, suggesting an elevated risk of HLH with trimethoprim/sulfamethoxazole; and (iii) at least three reported cases of HLH.^18,19^ Furthermore, adjusted RORs were calculated using logistic regression, with covariates including age category and respiratory and urinary tract infection indications. We adjusted for respiratory and urinary tract infection indications to address potential confounding by indication in spontaneous reporting. These infections are common reasons for prescribing trimethoprim/sulfamethoxazole and comparator antibiotics such as amoxicillin/clavulanate and azithromycin. All statistical analyses were conducted using SAS (Version 9.4) and R (Version 4.4.1).

## Results

### Literature review

A total of 1197 studies were identified through Medline and Embase searches. After removing 790 duplicates, 407 studies were screened. Among these studies, only five case reports were identified as potentially relevant, each describing an association between HLH and trimethoprim/sulfamethoxazole (Figure S1). No cross-sectional studies, cohort studies, or randomized controlled trials investigating this AE were identified.

The five reported cases involved patients with a median age of 34 years (IQR: 28.5–49), mostly male (4/5, 80%). HLH symptoms emerged a median of 9 days (IQR: 2.5–13) after trimethoprim/sulfamethoxazole initiation. Following HLH onset, all five patients (100%) discontinued trimethoprim/sulfamethoxazole, with symptom resolution occurring within a median of 24 hours (IQR: 2.5–13 days) after cessation. All cases reported symptoms, including fever, pancytopenia and elevated inflammatory markers. Few studies reported hyperferritinemia (60%), rash (60%), hypertriglyceridemia (40%), acute kidney injury (40%), liver dysfunction (40%) and coagulopathy (40%). Some cases reported progressive lethargy (20%), severe encephalopathy (20%), multiple organ failure (20%) and cervical lymphadenopathy (20%) (Table 1).^20–24^ The trimethoprim/sulfamethoxazole dosages varied across cases, including prophylactic and therapeutic regimens.

**Table 1.** Case report characteristics included in the review

Author/publication year	Age	Sex	Indication	Dose	Time to adverse event	Treatment	Outcome	Naranjo score^a^
Stark, 2023^20^	45	Male	Prophylaxis for hairy cell leukaemia	960 mg, 3 times/week	14 days after	Discontinuation of SXT, commenced on dexamethasone (10 mg/m^2^)	Clinical improvement; discharged on tapering steroid regimen; full recovery of blood counts within 2–3 months	3
Zimilover, 2022^21^	early 30s	Male	Perirectal abscess	7 day course	2 days after	IV dexamethasone (10 mg/m^2^)	Clinical improvement with normal laboratory markers; discharged on a steroid taper	3
Markiewicz, 2017^22^	34	Male	Sinusitis	N/A	3 days after	Discontinuation of SXT, corticosteroids on day 4 of hospitalization	Resolved multiple organ failure, neurologically intact; physical therapies upon ICU transfer, bone marrow not yet fully recovered	2
Lambotte, 2022^23^	53	Female	Pneumocystis carinii	960 mg/day (TMP) & 4800 mg/day (SMX); 6 double-strength tablets daily	9 days after	Discontinuation of SXT, switched to atovaquone	Apyrexia within 48 hours, clinical improvement, resolved biological abnormalities	6
Vernier, 2013^24^	25	Male	Pubic symphysis osteitis	1600 mg (sulfamethoxazole), 320 mg (trimethoprim), 3 times/day	12 days after	Discontinuation of SXT	Complete resolution of fever and normalisation of biological parameters within 10 days	6

SXT, trimethoprim/sulfamethoxazole.

^a^The Naranjo Scale was used for the quality score for the case reports. Score between 2 and 4 = possible ADR, 5 to 8 = probable ADR

The Naranjo scale was applied to assess causality. Three cases were classified as Possible Adverse Drug Reactions (ADRs), while two were deemed Probable ADRs (Table 1), reinforcing a potential link between trimethoprim/sulfamethoxazole and HLH. Treatment strategies included discontinuation of trimethoprim/sulfamethoxazole, initiation of corticosteroids and supportive care, with all patients achieving clinical improvement.

### Clinical characteristics of cases of HLH in FAERS

This analysis included 42 588 ICSRs: 10 067 for trimethoprim/sulfamethoxazole, 11 086 for amoxicillin/clavulanate and 21 435 for azithromycin (Figure S2). HLH was reported in 39 reports (0.09%) for trimethoprim/sulfamethoxazole, 14 (0.03%) for amoxicillin/clavulanate and 6 (0.01%) for azithromycin (Table 2). HLH-related reports for trimethoprim/sulfamethoxazole primarily occurred in younger patients (69.2% in individuals ≤18 years). Males were disproportionately affected (75.7% of trimethoprim/sulfamethoxazole reports). Geographically, 65.8% of trimethoprim/sulfamethoxazole-related HLH reports originated from the United States. Among trimethoprim/sulfamethoxazole-related HLH reports, 18.0% resulted in hospitalization, 2.6% were classified as life-threatening and no fatalities were reported.

**Table 2.** Clinical characteristics of reports of HLH associated with trimethoprim/sulfamethoxazole, amoxicillin/clavulanate and azithromycin from the FAERS database from 2004Q1 to 2023Q4

Characteristics	Trimethoprim/Sulfamethoxazole (N, %)	Amoxicillin/Clavulanate (N, %)	Azithromycin (N, %)
Number of reports of HLH	39 (0.09)	14 (0.03)	6 (0.01)
Age (year)			
Median age (IQR)	16 (7 to 20)	7 (7 to 29)	10 (7 to 17)
≤18	27 (69.2)	9 (64.3)	5 (83.3)
18–56	10 (25.6)	3 (21.4)	1 (16.7)
≥ 56	2 (5.1)	2 (14.3)	0 (0)
Sex			
Male	28 (75.7)	3 (21.4)	5 (83.3)
Female	9 (24.3)	11 (78.6)	0 (0)
Unknown	0 (0)	0 (0)	1 (16.7)
Indication of use			
UTI	1 (2.6)	2 (14.3)	0 (0)
RTI	0 (0)	2 (14.3)	0 (0)
Reported person			
Physician	12 (31.6)	1 (7.1)	1 (16.7)
Pharmacist	3 (7.9)	1 (7.1)	0 (0)
Others^a^	23 (60.5)	12 (85.7)	5 (83.3)
Unknown	1 (2.6)	0 (0)	0 (0)
Reporting country			
United States	25 (65.8)	0 (0)	1 (16.7)
Canada	7 (18.4)	9 (64.3)	0 (0)
Others	5 (12.8)	5 (35.7)	5 (83.3)
Unknown	1 (2.6)	0 (0)	0 (0)
Serious outcome			
Death	0 (0)	0 (0)	0 (0)
Hospitalisation	7 (18.0)	3 (21.4)	0 (0)
Life-threatening	1 (2.6)	0 (0)	0 (0)
Not serious	0 (0)	0 (0)	0 (0)
Other	31 (79.5)	11 (78.6)	6 (100.0)

FAERS, U.S. Food & Drug Administration Adverse Event Reporting System; IQR, interquartile; RTI, respiratory tract infection; UTI, urinary tract infection.

^a^Others include other healthcare professionals and consumers or non-health professionals.

### Active-comparator restricted disproportionality analyses in FAERS

In the ACR-DA of FAERS, trimethoprim/sulfamethoxazole was associated with a significantly higher frequency of adverse event reports than amoxicillin/clavulanate and azithromycin. A safety signal was identified for trimethoprim/sulfamethoxazole relative to amoxicillin/clavulanate (ROR, 3.08; 95% CI, 1.67–5.68; PRR, 3.07; 95% CI, 1.67–5.65; IC₂₅, 0.13). Similarly, trimethoprim/sulfamethoxazole showed markedly higher odds of HLH reports compared to azithromycin (ROR, 13.89; 95% CI, 5.88–32.82; PRR, 13.80; 95% CI, 5.86–32.7; IC₂₅, 0.92). These findings remained consistent in analyses adjusted for age and indications (Table 3), reinforcing the disproportionate association between trimethoprim/sulfamethoxazole and HLH compared to the comparator antibiotics.

**Table 3.** ROR, aROR, PRR with 95% CI, IC, and IC_25_ of haemophagocytic lymphohistiocytosis reports for trimethoprim/sulfamethoxazole in comparator analyses in FAERS

Comparator	ROR (95% CI)	aROR (95% CI) ^a^	PRR (95% CI)	IC (IC_25_)
Amoxicillin/clavulanate	3.08 (1.67–5.68)	3.44 (1.85–6.39)	3.07 (1.67–5.65)	0.62 (0.13)
Azithromycin	13.89 (5.88–32.82)	17.69 (7.44–42.05)	13.80 (5.86–32.7)	1.41 (0.92)

aROR, adjusted reported odds ratio; CI, confidence interval; IC, information component. PRR, proportional reporting ratio; ROR, reported odds ratio.

^a^Adjusted by age category, urinary tract infection and respiratory tract infection indication.

## Discussion

We conducted a comprehensive literature review and DA using the FAERS database to investigate the potential relationship between trimethoprim/sulfamethoxazole and HLH. Our review identified five case reports where HLH onset occurred within 2 to 14 days of trimethoprim/sulfamethoxazole initiation, predominantly in adult male patients. The case reports showed favourable outcomes compared with what is typically expected for HLH. These findings align with existing literature suggesting that medication-associated HLH, particularly when identified early and managed by discontinuing the causative drug, often follows a milder clinical course and has a better prognosis than HLH associated with genetic or malignant causes.^25^ These findings should be interpreted cautiously due to the limited sample size, short follow-up periods and potential gaps in data regarding other contributing factors.

Our DA revealed that reports of HLH associated with trimethoprim/sulfamethoxazole were 3 to 14 times more prevalent than those linked to comparator antibiotics like amoxicillin/clavulanate and azithromycin. These findings align with the Health Canada report suggesting a possible link between trimethoprim/sulfamethoxazole and HLH.

We observed a discrepancy in the age distribution of HLH cases associated with trimethoprim/sulfamethoxazole, with most FAERS reports involving paediatric patients, whereas published case reports primarily described young adults. This difference may reflect variations in clinical recognition and reporting practices. HLH is more commonly diagnosed in children due to stronger clinical suspicion and known genetic predispositions, which may lead to more frequent reporting in this group.^26^ In contrast, case reports in the literature may be subject to publication bias, favouring rare or unexpected presentations such as HLH in adults. These factors are likely to contribute to the observed differences in age distributions across the data sources.

Trimethoprim/sulfamethoxazole is known for common AEs such as photosensitivity, rash, gastrointestinal issues and haematologic effects. However, the emerging concern regarding HLH is particularly alarming.^27^ Our review adds to the growing evidence suggesting a possible link between trimethoprim/sulfamethoxazole and HLH, emphasizing the need for increased vigilance given the widespread use of this medication.

The proposed mechanism behind trimethoprim/sulfamethoxazole-induced HLH may involve a hypersensitivity reaction, triggering an exaggerated immune response and cytokine storm. Sulfamethoxazole metabolism could generate reactive metabolites that bind to proteins, activating cytotoxic T-cells and macrophages and producing excessive cytokines.^28^ Additionally, complicating factors such as pre-existing infections, genetic predispositions (e.g. PRF1 mutations) and immune dysfunction exacerbated by trimethoprim/sulfamethoxazole further complicate risk assessments.^29,30^ The oxidative stress induced by trimethoprim/sulfamethoxazole metabolism may also worsen immune dysregulation and inflammation.^31^ Healthcare professionals should be aware of these potential AEs and monitor patients closely, particularly those with pre-existing risk factors.

HLH has also been reported in association with several other drugs beyond trimethoprim/sulfamethoxazole, including non-antibiotics such as lamotrigine (an antiepileptic agent) and allopurinol (a xanthine oxidase inhibitor).^32^ Rare case reports have also implicated combinations of drugs such as haloperidol and quetiapine, sometimes involving substances like cocaine. Several antibiotics have also been implicated in case reports of HLH, including piperacillin-tazobactam, minocycline and rifampin.^33^ These reports suggest that drug-induced HLH is not unique to trimethoprim/sulfamethoxazole and may be associated with other broad-spectrum antimicrobials.^34^

Our study had many strengths. The in-depth literature review identified many cases of trimethoprim/sulfamethoxazole inducing HLH. An active comparator analysis allowed us to directly compare trimethoprim/sulfamethoxazole to other antibiotics, reducing the risk of spurious associations. We also utilised the information component (IC), which confirms the safety signal and enhances the robustness of our analysis. Combining case reports from the literature review with signals identified from the FAERS database, we presented the most comprehensive data available to date on the link between trimethoprim/sulfamethoxazole and HLH.

Our study had several limitations. First, using self-reported data from the FAERS database may have introduced data quality bias, as inaccuracies or inconsistencies in reporting could affect the validity of the findings. Second, notoriety bias could have influenced our results, as adverse events associated with widely recognised drugs such as trimethoprim/sulfamethoxazole may be reported more frequently than those related to less widely recognised medications. The heightened awareness due to Health Canada’s warning about the potential risk of HLH with trimethoprim/sulfamethoxazole may have led healthcare professionals to report such cases more frequently than cases involving other antibiotics if they were aware of this signal. This could introduce a reporting bias, where trimethoprim/sulfamethoxazole-associated HLH cases are more likely to be identified and reported than similar events caused by other medications. Third, co-prescription bias may have played a role, as trimethoprim/sulfamethoxazole is often prescribed alongside other medications, complicating the determination of whether HLH was truly induced by trimethoprim/sulfamethoxazole or by drug interactions, even though we restricted our analysis to reports listing trimethoprim/sulfamethoxazole or other study antibiotics as the primary suspect. Confounding bias remains a concern, as unmeasured variables such as underlying conditions, previous medication use and genetic factors could have influenced the outcomes, potentially masking or exaggerating the relationship between trimethoprim/sulfamethoxazole and HLH, even though we compared reports of HLH with trimethoprim/sulfamethoxazole to other antibiotics used for similar indications. Finally, our literature review identified only case reports linking trimethoprim/sulfamethoxazole to HLH. Although this was the most comprehensive evidence available to date, it offers a low level of certainty. These limitations highlight the need for further research using more robust data and experimental designs to better assess the relationship between trimethoprim/sulfamethoxazole and HLH.

Our findings suggest a potential association between trimethoprim/sulfamethoxazole use and an increased risk of HLH. Despite the inherent limitations of spontaneous reporting databases and the studies included in our review, our findings provide valuable insights and establish a basis for future research on the safety profile of trimethoprim/sulfamethoxazole. Additional pharmacoepidemiologic studies are necessary to confirm these results.

## Supplementary Material

dkaf231_Supplementary_Data

## Data Availability

The data that support the findings of this study are publicly available at https://open.fda.gov

## Supplementary data

Figures S1 and S2, Tables S1 to S3 are available as Supplementary data at *JAC* Online.
