# Supplementary material for: Trimethoprim/sulfamethoxazole and risk of haemophagocytic lymphohistiocytosis (HLH): a literature review and disproportionality analysis using individual case safety reports from FAERS
Source: J Antimicrob Chemother. 2025 Jul 15;80(9):2421–7. doi: 10.1093/jac/dkaf231 (PMC12404721; doi:10.1093/jac/dkaf231)
Supplement: dkaf231_Supplementary_Data [file dkaf231_supplementary_data.docx]

**Supplementary Materials For:**

**Trimethoprim/Sulfamethoxazole and Risk of Hemophagocytic Lymphohistiocytosis (HLH): A Literature Review and Disproportionality Analysis Using Individual Case Safety Reports from FAERS**

Rinko LAU^1^, Hasti SADEGHI^2^, Fatemeh AHMADI^3,4^, Niaz CHALABIANLOO^1^, Rebecca PREYRA^1^, Mohammad Ali OMRANI^1^, Flory T MUANDA^1,3,4,5*^

^1^ Department of Physiology and Pharmacology, Western University, London, Ontario, Canada,

^2^ Department of Biology, Western University, London, ON, Canada,

^3^ Department of Epidemiology & Biostatistics, Western University, London, Ontario, Canada

^4^ ICES Western, London, Ontario, Canada,

^5^ Lawson Health Research Institute, London Health Sciences Centre, London, Ontario, Canada

**Corresponding Author**: Dr. Flory T Muanda, Department of Physiology and Pharmacology, Medical Sciences Building, 1151 Richmond St, Room 287, London, Ontario, Canada N6A 5C1 Tel: 519-661-2111-extension 84885,

Email: [fmuandat@uwo.ca](mailto:fmuandat@uwo.ca)

**Running title**: Trimethoprim/Sulfamethoxazole and Risk of Hemophagocytic Lymphohistiocytosis

**Data availability statement**: The data that support the findings of this study are publicly available at [https://open.fda.gov](https://open.fda.gov/)

***Table S1.*** Literature search strategy

| **Database** | **Search Strategy** |
| --- | --- |
| **Medline** | 1. (sulfamethoxazole or trimethoprim or co?trimoxazole or SMZ-TMP or TMP-SMX or TMP-SMZ or SMX-TMP).mp. |
|  | 2. ((H?emophagocytic adj2 Lympho?histiocytosis) or (erythrophagocytic adj2 lymphohistiocytosis) or h?emophagocytic syndrome? or HLH).mp. |
|  | 3. exp Trimethoprim, Sulfamethoxazole Drug Combination/ae, po, to [Adverse Effects, Poisoning, Toxicity] |
|  | 4. 1 or 3 |
|  | 5. 2 and 4 |
| **Embase** | 1. (sulfamethoxazole or trimethoprim or co?trimoxazole or SMZ-TMP or TMP-SMX or TMP-SMZ or SMX-TMP).mp. |
|  | 2. ((H?emophagocytic adj2 Lympho?histiocytosis) or (erythrophagocytic adj2 lymphohistiocytosis) or h?emophagocytic syndrome? or HLH).mp. |
|  | 3. exp cotrimoxazole/ae, to, tm [Adverse Drug Reaction, Drug Toxicity, Unexpected Outcome of Drug Treatment] |
|  | 4. 1 or 3 |
|  | 5. 2 and 4 |

***Table S2.* The READUS-PV checklist for abstracts**

| **Section and topic** | **Item #** | **Checklist item** | **Location where item is reported** |
| --- | --- | --- | --- |
| Background | *1a* | *State the aim/rationale for performing the study.* | Page 2 |
|  | *1b* | *Specify the adverse event(s) and/or the drug(s) under study, when applicable.* | Page 2 |
|  | *1c* | *Specify the specific population or setting, when applicable.* | Page 2 |
| Methods | *2a* | *Identify the study as a “disproportionality analysis” and specify the type of data used.* | Page 2 |
|  | *2b* | *Specify the name of the database(s) used and the type of access.* | Page 2 |
|  | *2c* | *Specify the timeframe and geographical region, when applicable.* | Page 2 |
|  | *2d* | *Specify the disproportionality measure(s) used and their statistical significance threshold(s).* | Page 2 |
|  | *2e* | *Specify if a case-by-case analysis is performed.* | Page 2 |
| Results | *3* | *Report main findings including their precision (e.g., 95% confidence intervals), together with a short summary of the case-by-case analysis.* | Page 2 |
| Conclusion | *4a* | *Clearly report key conclusions.* | Page 2 |
|  | *4b* | *Acknowledge that the disproportionality analysis is a hypothesis generating or refinement approach.* | Page 2 |
|  | *4c* | *State the implications and clinical relevance of the findings.* | Page 2 |

***Table S3.*** **The READUS-PV checklist**

| **Section and topic** | **Item #** | **Checklist item** | **Location where item is reported** |
| --- | --- | --- | --- |
| **Title** |  |  |  |
|  | *1a* | *If disproportionality analyses are a prominent component of the published study, the study should be identified as a “disproportionality analysis”. The type of data and name of the database(s) should be specified.* | Page 1 |
|  | *1b* | *Report the name of adverse event(s) and/or drug(s) under study, when applicable.* | Page 1 |
| **Introduction** |  |  |  |
| Background | *2a* | *Describe the drug(s) and its utilization, the nature of the adverse event(s) under study and its frequency, and the existing knowledge on the drug-event combination.* | Page 3 |
|  | *2b* | *Specify the rationale for performing the analysis, e.g., as part of routine pharmacovigilance, to investigate an overall safety profile, or to assess a pre-specified hypothesis.* | Page 3 |
|  | *2c* | *Explain why ICSR databases and disproportionality analysis are suitable to fill the knowledge gap.* | Page 3 |
| Objectives | *3* | *State specific objectives, identifying the adverse event(s), the drug(s), and the reference group, including any pre-specified hypothesis, if applicable.* | Page 4 |
| **Methods** |  |  |  |
| Study design | *4a* | *Identify the study (i.e., “disproportionality analysis”) and the type of data used (e.g., “individual case safety reports”).* | Page 4-5 |
|  | *4b* | *Provide an outline of the entire study design, including primary and sensitivity analyses performed, and other designs such as case-by-case analysis or literature review.* | Page 4-5 |
| Data description, access, and pre-processing | *5a* | *Specify the name of the database(s), the database(s) custodian, and the coverage. Specify the type/number of drugs included within the database and the thesaurus, taxonomies, or ontologies used for coding drugs and events.* | Page 4-5 |
|  | *5b* | *Specify the extraction dates and describe and justify all choices used for data pre-processing, including any data transformation or exclusion, if appropriate.* | Page 5 |
| Variables definition | *6a* | *Describe the study population, including any restriction.* | Page 6 |
|  | *6b* | *Describe the nature and the meaning of key variables assessed in the work.* | Page 6 |
|  | *6c* | *Specify and justify any grouping of drugs or events. For drugs, specify and justify whether active ingredients/trade names/salts were considered and/or the selected role.* | Page 6 |
|  | *6d* | *Describe any additional data source used, the type of data, and how they interact with ICSRs.* | Page 6 |
| Statistical methods | *7a* | *Present any descriptive analysis performed, specifying variables investigated, statistical tests, and significance thresholds.* | Page 6 |
|  | *7b* | *Describe the measure(s) selected for the disproportionality analysis including any threshold used to identify signals of disproportionate reporting. Explain the reason for this choice if applicable.* | Page 6 |
|  | *7c* | *Clearly describe any sensitivity analysis and any tool to control confounding, including any restriction, subgroup, stratification, adjustment, or interaction.* | Page 6 |
|  | *7d* | *Specify the variables and methods used for the case-by-case analysis, including any algorithm or criteria used to assess causality, if performed.* | Page 6 |
|  | *7e* | *Specify any statistical methods used for other data sources.* | Page 6-7 |
| **Results** |  |  |  |
| Participants | *8a* | *Specify the number of individual case safety reports included at each stage, including reasons for exclusion.* | Page 7 |
|  | *8b* | *Provide key demographic and clinical characteristics of cases, if possible comparing cases with any appropriate reference group.* | Page 7-8 |
| Disproportionality analysis | *9* | *Present all results including confidence intervals. Present also results of sensitivity analyses, if performed.* | Page 7-8 |
| Case-by-case analysis | *10* | *Present the case-by-case analysis of key variables. Present the causality assessment, if applicable.* | Page 7-8 |
| **Discussion** |  |  |  |
| Key results | *11* | *Discuss key results with reference to study objectives and contextualize them within the current literature and other consulted sources. Clearly discriminate between expected reactions and emerging safety signals.* | Page 8-9 |
| External validity | *12a* | *Discuss the external validity of the results to the general population.* | Page 8-9 |
|  | *12b* | *Discuss the potential relevance of results in clinical practice* | Page 8-10 |
|  | *12c* | *Propose further study designs if applicable* | Page 9-10 |
| Limitations | *13* | *Present general limitations, making clear that disproportionality analysis alone cannot prove causation or measure incidence, and specific limitations, including confounding and reporting bias and efforts to mitigate them.* | Page 10 |
| **Declarations** |  |  |  |
|  | *14a* | *Provide the source of funding/sponsorship and the role of the funders/sponsors for the present study and for any original study on which the present article is based.* | Page 11 |
|  | *14b* | *Clearly identify potential commercial and intellectual conflicts of interest (e.g., link to any drug/event investigated, whether financial, legal action, or software used).* | Page 11 |
|  | *14c* | *Declare any institutional approval needed or granted in the investigation.* | Page 11 |
|  | *14d* | *Include a statement on data availability, code availability (including the version of the statistical software used), and protocol registration.* | Page 11 |

**Figure S1**. Literature Review Prisma Flowchart

Studies from databases/registers **(n = 1197)**

Embase (n = 1114)

MEDLINE (n = 82)

References from other sources **(n = 1)**

Included studies ongoing **(n = 0)**

Studies awaiting classification **(n = 0)**

Studies included in review **(n = 5)**

Studies excluded **(n = 399)**

Studies not retrieved **(n = 0)**

Studies assessed for eligibility **(n = 8)**

Studies sought for retrieval **(n = 8)**

Studies screened **(n = 407)**

Studies excluded **(n = 3)**

Wrong outcomes (n = 1)

Wrong study design (n = 2)

**Identification**

References removed **(n = 790)**

Duplicates identified manually (n = 0)

Duplicates identified by Covidence (n = 790)

Marked as ineligible by automation tools (n = 0)

**Screening**

**Included**

**Figure S2**. Flow Diagram
